# Valiltramiprosate/ALZ‐801 Prevents Hippocampal Atrophy and Clinical Decline in a Stage 2 AD Subpopulation in APOLLOE4 Phase 3 Study

**DOI:** 10.1002/alz70861_108565

**Published:** 2025-12-23

**Authors:** Jeremy Yu, Hugo Geerts, Shaina M Short, Jean Schaefer, Susan Abushakra, Aidan Power, Martin Tolar, John A. Hey

**Affiliations:** ^1^ Alzheon, Framingham, MA USA; ^2^ Certara Predictive Technologies, Berwyn, PA USA; ^3^ Certara Predictive Technologies, Park City, UT USA; ^4^ Alzheon, Inc., Framingham, MA USA

## Abstract

**Background:**

The central hallmark of Alzheimer's disease (AD) is progressive brain atrophy accompanied by amyloid and tau pathologies. Valiltramiprosate is an oral, small‐molecule inhibitor of amyloid oligomer formation in development as a disease‐modifying treatment for AD. Whereas APOLLOE4 did not meet the primary endpoint of cognitive slowing in overall Early AD population, valiltramiprosate produced a meaningful improvement (ΔADAS‐Cog13 = ‐2.144, *p* =0.041) and function (ΔCDR‐SB = ‐0.646, *p* =0.053) in the prespecified Mild Cognitive Impairment (MCI) subjects. Valiltramiprosate consistently reduced atrophy in hippocampus (‐26%, *p* =0.0042 in MCI) and other brain regions, indicating substantive neuroprotective benefits. We report a subpopulation of early symptomatic super‐responders in the APOLLOE4 Phase 3 study that exhibited full prevention of hippocampal atrophy and improved cognition over 78 weeks.

**Method:**

The multicenter, randomized, double‐blind, placebo‐controlled APOLLOE4 Phase 3 trial was conducted in 325 APOE4/4 homozygotes with MCI or Mild AD (MMSE ≥22 and CDR‐Global 0.5 ‐ 1.0). The valiltramiprosate dose was 265 mg BID for 78 weeks. The primary outcome was ADAS‐Cog13, and volumetric hippocampal volume (HV) MRI was an imaging endpoint. The subpopulation reflects AD subjects with an increased HV at 78 weeks over baseline.

**Result:**

Eleven super‐responder subjects (MMSE 27.7 ± 0.6; 6 males and 5 females) in the active treatment group and two female placebo subjects (MMSE 28.0 ± 0.0) exhibited a greater HV at 78 weeks than baseline. This represents ∼15% and 6.5% of the active MCI and Mild AD subject populations, respectively. There was an increase of 66.0 ± 20.4 mm^3^ (mean ± SEM) HV at 78 weeks vs. baseline in the active super‐responders. Also, the active super‐responders exhibited a younger age (63.7 ± 2.4 years), higher MMSE, and less baseline hippocampal atrophy (8511.6 ± 348.7 mm^3^) compared to the overall Early AD population (7045 ± 1002 mm^3^). The prevention of atrophy by valiltramiprosate treatment corresponded with cognitive and functional benefit (ADAS‐Cog13, CDR‐SB).

**Conclusion:**

Valiltramiprosate demonstrates potential for preventing hippocampal atrophy and stabilization of cognitive and functional decline in earliest symptomatic APOE4/4 AD. The results support a potential disease stabilization benefit of oral valiltramiprosate as a prevention therapy in presymptomatic AD.

